# Magnetic resonance imaging-induced artefact mimicking torsade de pointes in a patient with an implantable cardiac monitor: a case report

**DOI:** 10.1093/ehjcr/ytag360

**Published:** 2026-05-13

**Authors:** Yasuyuki Toyama, Yusuke Taniguchi, Mayu Morisue, Yoshiaki Matsuo

**Affiliations:** Department of Cardiovascular Medicine, Iseikai International General Hospital, 4-14 Minamiogi-machi, Kita-ku, Osaka 530-0052, Japan; Department of Cardiovascular Medicine, Iseikai International General Hospital, 4-14 Minamiogi-machi, Kita-ku, Osaka 530-0052, Japan; Department of Clinical Engineering, Iseikai International General Hospital, 4-14 Minamiogi-machi, Kita-ku, Osaka 530-0052, Japan; Department of Clinical Engineering, Iseikai International General Hospital, 4-14 Minamiogi-machi, Kita-ku, Osaka 530-0052, Japan

**Keywords:** Magnetic resonance imaging, Implantable cardiac monitor, Torsade de pointes, Artefact, Electromagnetic interference, Case report

## Abstract

**Background:**

Magnetic resonance imaging (MRI) is increasingly performed in patients with cardiac implantable electronic devices. Although generally considered safe under specific protocols, electromagnetic interference may still lead to clinically misleading recordings.

**Case summary:**

A 76-year-old woman with a history of mitral valve replacement and paroxysmal atrial fibrillation underwent implantation of an implantable cardiac monitor (Abbott Confirm Rx) for the evaluation of recurrent presyncope. During a brain MRI at another hospital (1.5-T GE SIGNA Creator), the device captured high-amplitude polymorphic signals resembling torsade de pointes, whereas a 12-lead electrocardiogram obtained shortly after MRI confirmed sinus rhythm with a normal QT interval. A repeat MRI at our institution using a 3.0-T Siemens MAGNETOM Lumina scanner with the same time-of-flight magnetic resonance angiography sequence did not reproduce the artefact. Phantom testing on the outside hospital’s scanner also failed to demonstrate the phenomenon. However, the artefact was reproducibly observed during a follow-up MRI performed 6 months later using the same scanner. No similar findings were observed in other patients with implantable cardiac monitors scanned using the same system.

**Discussion:**

MRI-related artefacts in implantable loop recorders have been previously reported. The present case demonstrates a reproducible torsade de pointes-like morphology specifically associated with the pre-scan phase on a particular MRI system, suggesting a device–scanner–patient interaction rather than true arrhythmia.

Learning points

- MRI pre-scan sequences may reproducibly induce high-amplitude artefacts in implantable cardiac monitors mimicking torsade de pointes through device–scanner interactions.
- Correlation with peri-MRI surface electrocardiography, including assessment of the QTc interval, is essential to differentiating true arrhythmia from artefact and preventing inappropriate management.

## Introduction

Magnetic resonance imaging (MRI) is increasingly used in patients with implantable cardiac devices. Although safety has improved, electromagnetic interference can still cause artefacts in intracardiac electrograms.^1^ Such artefacts may mimic life-threatening arrhythmias. We report a reproducible case of torsade de pointes-like artefact during the prescan phase of time-of-flight magnetic resonance angiography, a routine calibration step including centre-frequency determination, B0 shimming, and radiofrequency calibration in a patient with an implantable cardiac monitor. With the widespread adoption of implantable cardiac monitors in recent years, the number of patients undergoing MRI examinations while carrying such devices has increased substantially. Recognition of potential artefacts in this setting is therefore of growing clinical importance.

## Summary figure

**Figure:**
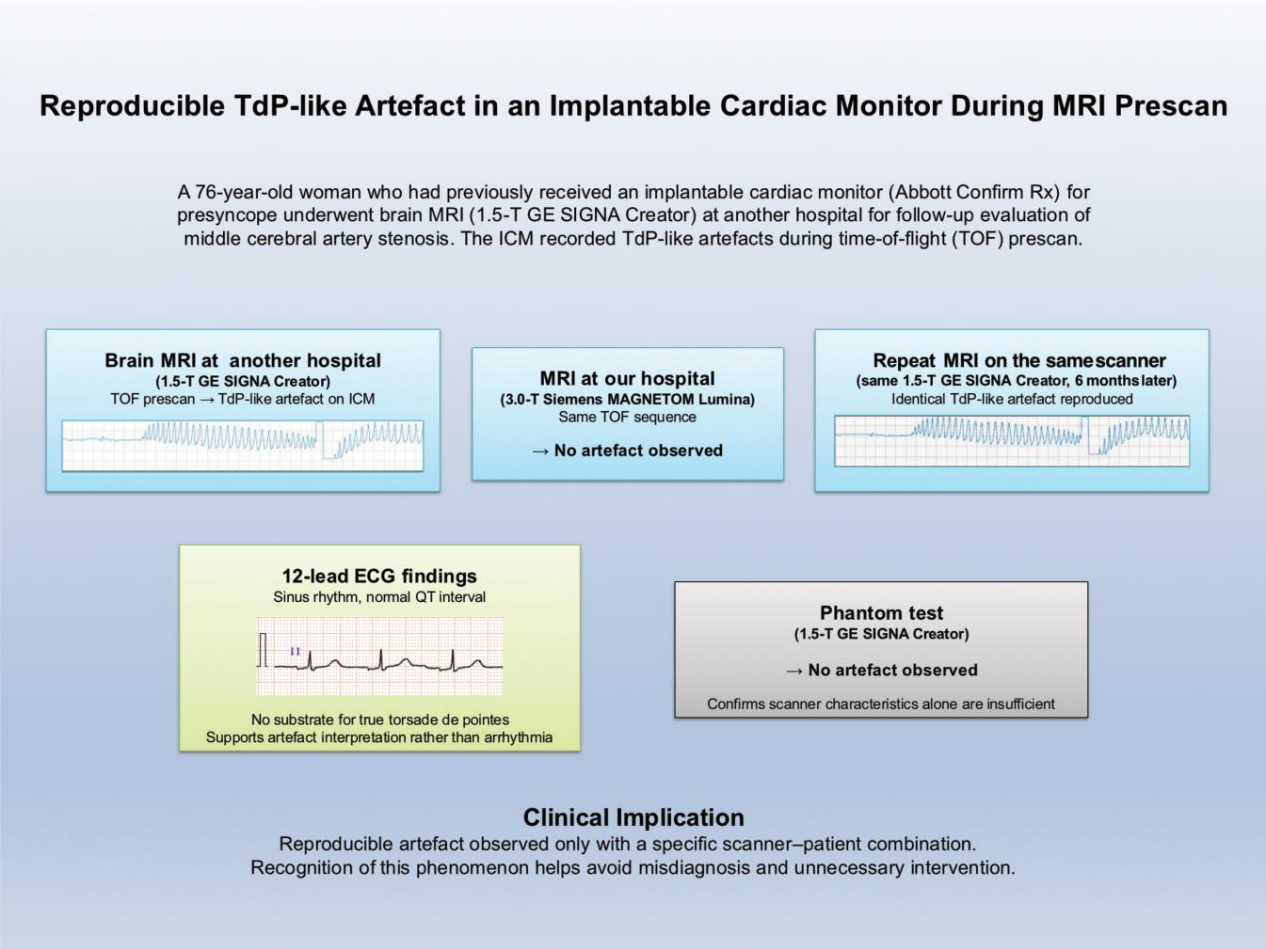


## Case presentation

A 76-year-old woman with a history of rheumatic valvular disease had undergone mitral valve replacement 2.5 years earlier. An implantable cardiac monitor (Abbott Confirm Rx) had been implanted for evaluation of recurrent presyncope (described as transient lightheadedness without complete loss of consciousness). She also had paroxysmal atrial fibrillation treated with a beta-blocker but had never received class I or class III antiarrhythmic agents. She was otherwise clinically stable, with no family history of sudden cardiac death.

**Figure 1:**
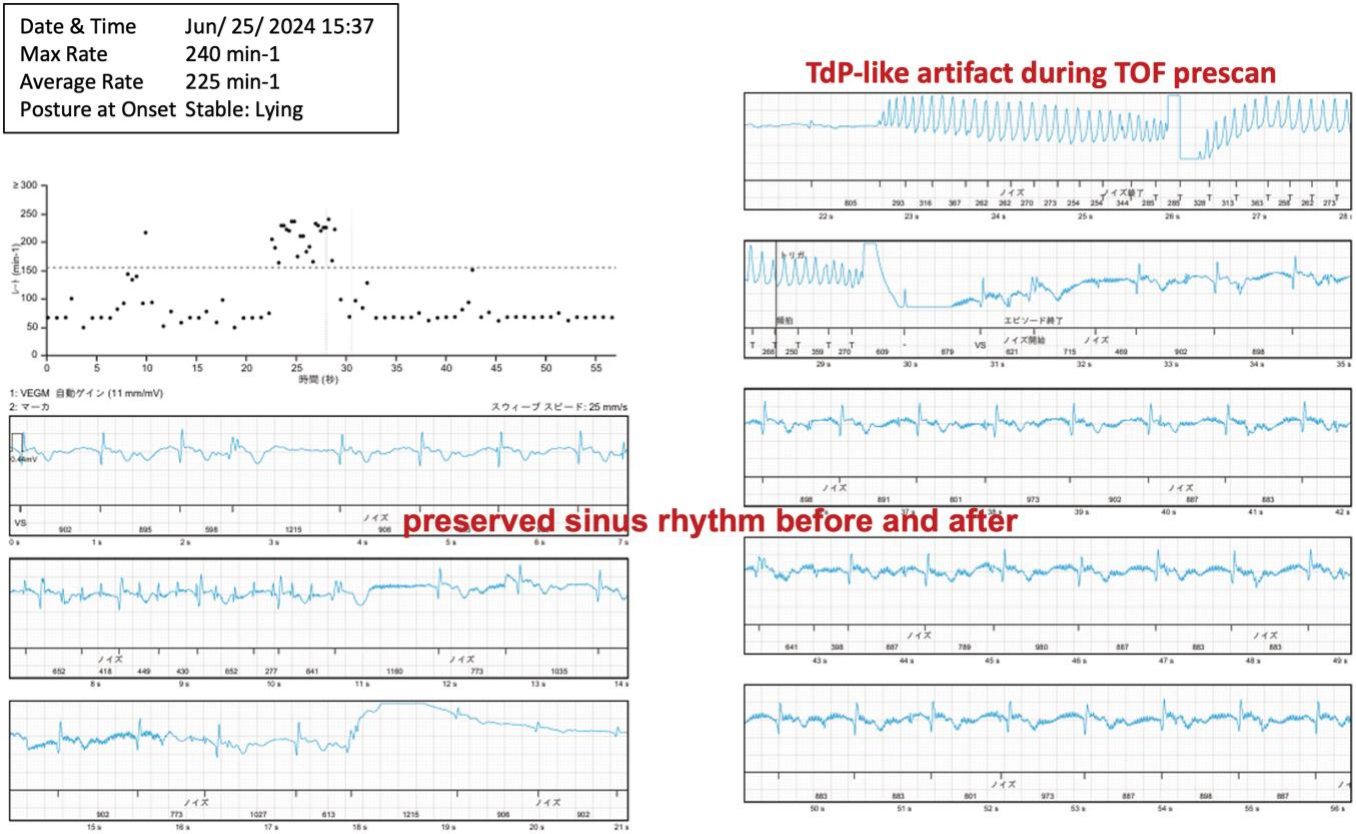
Implantable cardiac monitor (ICM) recording during brain magnetic resonance imaging (1.5-T GE SIGNA Creator) showing high-amplitude polymorphic signals mimicking torsade de pointes. The recording occurred during the pre-scan phase of time-of-flight magnetic resonance angiography. These signals were not associated with true arrhythmias.

**Figure 2:**
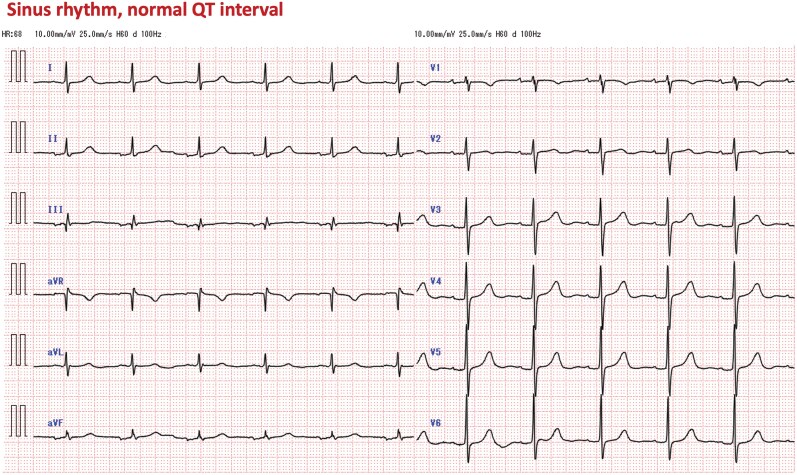
Twelve-lead electrocardiogram obtained after magnetic resonance imaging demonstrating sinus rhythm without polymorphic ventricular tachycardia. The corrected QT interval (QTc) was approximately 410 ms calculated using the Bazett formula, supporting an artefactual rather than arrhythmic origin of the ICM recording.

During brain MRI at another hospital (1.5-T GE SIGNA Creator), the implantable cardiac monitor recorded high-amplitude polymorphic signals resembling torsade de pointes (*Figure 1*). A 12-lead electrocardiogram obtained shortly after MRI confirmed sinus rhythm with a normal QT interval (*Figure 2*). The absence of QT prolongation provided strong supportive evidence that the abnormal tracing represented an artefact rather than a true arrhythmia.

**Figure 3:**
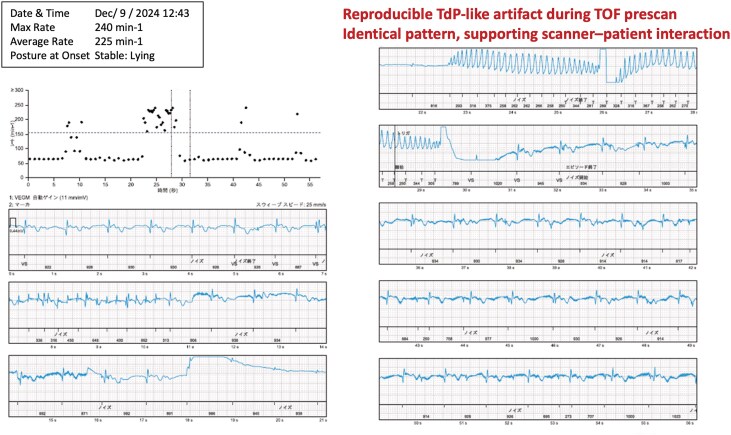
Reproducible ICM recording obtained during follow-up magnetic resonance imaging performed 6 months later using the same 1.5-T scanner. Similar high-amplitude polymorphic signals were observed during the pre-scan phase, demonstrating reproducibility of the artefact. No comparable findings were observed on a different MRI system or during phantom testing.

The patient was subsequently referred to our institution. Repeat MRI performed using a 3.0-T Siemens MAGNETOM Lumina scanner with the same time-of-flight magnetic resonance angiography sequence did not reproduce the abnormal recordings. Phantom testing performed at the outside hospital on the 1.5-T scanner also failed to demonstrate abnormal recordings. However, at the scheduled 6-month follow-up, repeat MRI on the same scanner again induced an identical torsade de pointes-like recording during the pre-scan phase (*Figure 3*). In other patients implanted with implantable cardiac monitors and scanned using the same MRI system, no such artefacts were detected.

Throughout all MRI sessions, the patient remained asymptomatic without palpitations, chest pain, or syncope. No device reprogramming or antiarrhythmic therapy was required. At the 12-month follow-up, she continued to do well without recurrence of suspicious recordings outside MRI examinations.

## Discussion

Correlation with surface electrocardiography is indispensable for accurate interpretation. Documentation of a normal QT interval provided the most convincing evidence that the torsade de pointes-like recordings were artefacts, since a prolonged QT interval is generally required for the initiation of true torsade de pointes.

Pre-scan calibration procedures, such as B0 shimming and radiofrequency transmit calibration, may transiently couple electromagnetic interference into the sensing circuit.^2^

The absence of the artefact on the 3.0-T Siemens scanner, despite its higher magnetic field strength, suggests that field strength alone does not determine the occurrence of this phenomenon. Instead, the finding points towards differences in vendor-specific pre-scan implementations. Pre-scan calibration processes—such as centre-frequency determination, B0 shimming, and radiofrequency power calibration—are executed differently across MRI systems and may involve distinct gradient switching patterns, RF pulse characteristics, and timing structures. These differences can generate transient electromagnetic environments that interact variably with the sensing circuitry of implantable cardiac monitors.

In this context, the reproducible occurrence of the artefact on the same 1.5-T scanner, but not on the 3.0-T system using a nominally similar imaging sequence, supports the hypothesis that scanner-specific pre-scan architecture, rather than magnetic field strength *per se*, plays a central role. However, because the individual pre-scan components were not separately isolated or analysed in this clinical setting, it was not possible to determine whether a specific step—such as B0 shimming, centre-frequency determination, or RF calibration—was primarily responsible. This limitation should be acknowledged when interpreting the mechanistic implications of this observation.

The reproducibility of the artefact in this patient, but not in others scanned using the same MRI system and sequence, suggests that scanner-related factors alone are insufficient. Patient-dependent factors, including thoracic anatomy, tissue conductivity, and device position, may potentially modify electromagnetic field distribution during pre-scan calibration and contribute to oversensing. In the present case, the implantable cardiac monitor was positioned in the standard left parasternal region without an abnormal device configuration. Although phantom testing failed to reproduce the phenomenon, this likely reflects the absence of patient-specific conductive and anatomical characteristics.

The device manufacturer was consulted, and no device malfunction or programming abnormality was identified. Sensing parameters remained unchanged before and after MRI examinations, further supporting the interpretation of a device–scanner interaction rather than intrinsic device failure. All MRI examinations were performed in accordance with established safety protocols for patients with cardiac implantable electronic devices.

From a clinical perspective, misinterpretation of such recordings as true arrhythmia could lead to unnecessary antiarrhythmic therapy, inappropriate escalation of care, or unwarranted device implantation. We therefore recommend routine correlation of device electrograms with peri-MRI surface electrocardiography and heightened awareness of prescan-related artefacts among clinicians managing patients with implantable cardiac monitors. Previous reports have described electromagnetic interference in pacemakers and implantable cardioverter-defibrillators during MRI, particularly during pre-scan or gradient-intensive sequences, resulting in oversensing or pacing inhibition.^3–5^ Similar artefacts have also been reported in implantable loop recorders during MRI, including tachyarrhythmia- and bradyarrhythmia-like recordings.^6,7^ In contrast, evidence regarding implantable cardiac monitors remains scarce. Recognition of this benign yet deceptive phenomenon is essential to avoid misdiagnosis and ensure appropriate patient management.

From a practical clinical perspective, a torsade de pointes-like tracing detected on an implantable cardiac monitor during MRI should be interpreted within a structured framework. First, the temporal relationship between the recording and the MRI examination should be assessed, particularly whether the event occurs during the pre-scan phase or image acquisition. Second, the presence or absence of symptoms should be carefully evaluated. Third, correlation with peri-MRI surface electrocardiography is essential, including confirmation of an underlying rhythm and assessment of the QTc interval. Finally, the reproducibility of the finding outside the MRI environment should be considered. Concordant sinus rhythm on surface ECG, absence of QT prolongation, and restriction of the phenomenon to the MRI setting strongly support an artefactual origin rather than true polymorphic ventricular tachycardia.

## Conclusion

MRI pre-scan sequences may reproducibly induce torsade de pointes-like artefacts in implantable cardiac monitors through device–scanner–patient interaction. Recognition of this phenomenon and correlation with surface electrocardiography can prevent misdiagnosis and inappropriate management.

## Data Availability

The data underlying this article are available within the article. Additional data are not publicly available due to patient privacy considerations.

## Lead author biography

Dr. Yasuyuki Toyama is an interventional cardiologist at Iseikai International General Hospital in Osaka, Japan. His clinical practice focuses on percutaneous coronary intervention and coronary physiology assessment. He has an interest in coronary microvascular dysfunction and is also involved in clinical education and multidisciplinary cardiovascular care.

## Notes

**Consent:** Written informed consent was obtained from the patient for publication of this case report, including clinical data and images, in accordance with COPE guidance.
